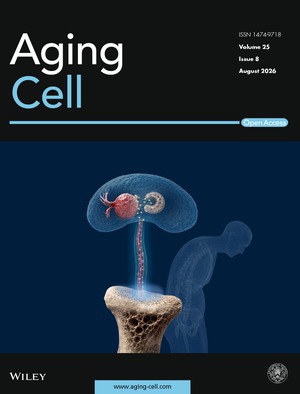# Featured Cover

**DOI:** 10.1111/acel.70665

**Published:** 2026-08-12

**Authors:** Dandan Zhong, Chang Hao, Mengyue Li, Jing Liu, Zheng Xu, Jianteng Zhou, Lu Zhao, Siyu Ni, Zhenchao Hu, Yue Sun, Yingying Zou, Dong Sun, Hao Guo, Zhanjun Jia, Dong Guo, Jun‐Li Cao, Ying Sun

## Abstract

Cover legend: The cover image is based on the article *Podocyte mPGES‐2 Determines Renal Aging and Contributes to Senile Osteoporosis* by Dandan Zhong et al., https://doi.org/10.1111/acel.70609.